# Evaluation of the Inhibitory Effects of *Lactobacillus gasseri* and *Lactobacillus crispatus* on the Adhesion of Seven Common Lower Genital Tract Infection-Causing Pathogens to Vaginal Epithelial Cells

**DOI:** 10.3389/fmed.2020.00284

**Published:** 2020-06-19

**Authors:** Yuanhui He, Xiaoxi Niu, Ben Wang, Risu Na, Bingbing Xiao, Huixia Yang

**Affiliations:** Department of Obstetrics and Gynecology, Peking University First Hospital, Beijing, China

**Keywords:** *Lactobacillus gasseri*, *Lactobacillus crispatus*, adhesion inhibition, lower genital tract infection-causing pathogens, vaginal epithelial cells

## Abstract

**Background/Purpose:** *Lactobacillus* colonization is important to maintain urogenital flora stability and prevent pathogenic infection. Different *Lactobacillus* species have distinct properties and effects on the urogenital flora. To select probiotics that colonize the vagina and provide protection against pathogenic infection, we evaluated the adhesion of five *Lactobacillus* strains and their inhibitory effects on the adhesion of pathogens to vaginal epithelial cells (VECs).

**Methods and Materials:** (1) *Lactobacillus* adhesion experiments: VK2/E6E7 and primary VECs were used to evaluate the adhesion of two *Lactobacillus gasseri* and three *Lactobacillus crispatus* strains. The adhesion of these five *Lactobacillus* strains was compared. (2) Adhesion inhibition experiments: The inhibitory effects of the five *Lactobacillus* strains on the adhesion of pathogens (*Gardnerella, Mobiluncus, Candida albicans, Streptococcus agalactiae, Staphylococcus aureus, Escherichia coli*, and *Enterococcus faecalis*) were evaluated by adhesion exclusion, displacement, and competition experiments.

**Results:** (1) *Lactobacillus* adhesion was stronger in the primary VECs than in the VK2/E6E7 VECs (*P* < 0.05). The adhesion of the three *L. crispatus* strains was stronger than that of the two *L. gasseri* strains (*P* < 0.05). *L. crispatus* 4# showed the strongest adhesion. (2) The exclusion, displacement, and competition experiments showed that all five *Lactobacillus* strains significantly inhibited the adhesion of the seven pathogenic strains to the VECs (*P* < 0.05). The displacement effect was stronger than the exclusion and competition effects of each *Lactobacillus* strain. (3) The results of the exclusion, displacement, and competition experiments indicated that *L. gasseri* 1# showed the strongest adhesion inhibition of *C. albicans* and *S. agalactiae*. *L. crispatus* 3# showed the strongest adhesion inhibition of *S. aureus*, whereas *L. crispatus* 4# showed the strongest adhesion inhibition of *Gardnerella, Mobiluncus, E. coli*, and *E. faecalis*.

**Conclusion:** The source of the VECs might not affect the selection of the most adhesive *Lactobacillus* strain. *L. crispatus* showed stronger VEC adhesion than *L. gasseri*. The degree of antagonism of the *Lactobacillus* strains toward the different pathogens varied. This result provides incentives for personalized clinical treatment.

## Introduction

Bacterial vaginosis (BV), vulvovaginal candidiasis (VVC), and aerobic vaginitis (AV) are common lower genital tract diseases that seriously affect female reproductive health (1–4). They are associated with infertility, ectopic pregnancy, and sexually transmitted diseases. *Gardnerella* and *Mobiluncus* are the most common causative pathogens of BV (5), whereas *Candida albicans* is the most common causative pathogen of VVC (6). AV is often associated with pathogens such as *Streptococcus agalactiae, Staphylococcus aureus, Escherichia coli*, and *Enterococcus faecalis* (7).

Antibiotics play an important role in the treatment and prevention of female reproductive tract infections, but their long-term use increases the rates of bacterial resistance and disease recurrence (8) and can severely disrupt the vaginal microbiota (9). Disease recurrence compromises the patient's quality of life and mental state (10). The active investigation of new methods for protection against pathogens is an important measure to prevent genital tract infections.

Various clinical studies have indicated that microecological preparations, administered orally or vaginally, can significantly reduce the incidence and recurrence rates (11–13), prolong the recurrence period, improve the recovery rate (14), relieve the symptoms (15), and improve the vaginal microecological patterns (16) of BV and VVC. There is a significant correlation between *Lactobacillus* colonization in the vagina and clinical outcomes (12, 13, 17). *Lactobacillus*, as the dominant bacterium, can balance the microbial flora of the genitourinary tract through a variety of mechanisms including host immune regulation, recovery of the vaginal flora, and interference with pathogen colonization (18, 19). It is important for restoring the normal state of the flora and preventing infections and disease recurrence (9, 18, 20, 21). Adhesion plays a key role in the beneficial effects of *Lactobacillus* (9) and is also the key virulence factor for pathogens such as *Gardnerella, C. albicans*, and *E. coli* (9).

It is of great importance to select better probiotic strains for clinical application. At present, more than 20 types of lactic acid-producing bacteria can be detected in the vagina by sequencing, and *Lactobacillus gasseri* and *Lactobacillus crispatus* are the main lactobacilli in healthy women (22–24). Their use can reduce the risk of the vigorous growth of non-native *Lactobacilli* in the urogenital tract (25). They produce antibacterial substances such as lactic acid and hydrogen peroxide (26, 27), adhere to vaginal epithelial cells (VECs) to form a protective film while inhibiting pathogen adhesion (26), and thus protect the vaginal epithelial barrier from pathogen colonization and invasion (28–30).

To select probiotics that could successfully colonize the vagina and protect it against pathogenic infection, we selected five *Lactobacillus* (two *L. gasseri* and three *L. crispatus*) strains, isolated from vaginal samples obtained from Chinese women, to evaluate their adhesion to the VECs and their ability to inhibit the adhesion of seven pathogens associated with common reproductive tract infections.

## Materials and Methods

### Origin and Culture of Bacterial Strains

Twelve archived and previously characterized clinical isolates of *Lactobacillus* and pathogenic strains (Table 1) were tested to determine their adhesion properties and the inhibitory effects of *Lactobacillus* on pathogen adhesion. The *Lactobacilli* were isolated from vaginal samples obtained from healthy volunteers (>18 years of age) at the health checkup clinic of our hospital. These women of childbearing age did not suffer from any urogenital tract infections. They had not used antibacterial drugs within 3 months of admission, and they had not had sexual intercourse 1 week before admission. The pathogens were isolated from patients (>18 years of age) with urogenital tract infections. All isolates were identified by standard methods used in clinical microbiology laboratories. Every strain to be tested was recovered and purified before the experiment to ensure bacterial viability and purity. The strains were incubated at 37°C in an atmosphere of 5% CO_2_ for 48 h. Each bacterial suspension was adjusted to a concentration of 1.0 × 10^8^ CFU/mL.

**Table 1:** The species, number, full name, and abbreviated name of the bacteria and vaginal epithelial cells used in this study.

**Species**	**Number**	**Full name**	**Brief name**
*Lactobacillus gasseri*	2	*Lactobacillus gasseri* 1#	*L. gasseri* 1#
		*Lactobacillus gasseri* 2#	*L. gasseri* 2#
*Lactobacillus crispatus*	3	*Lactobacillus crispatus* 3#	*L. crispatus* 3#
		*Lactobacillus crispatus* 4#	*L. crispatus* 4#
		*Lactobacillus crispatus* 5#	*L. crispatus* 5#
*Gardnerella*	1	*Gardnerella*	*Gardnerella*
*Mobiluncus*	1	*Mobiluncus*	*Mobiluncus*
*Candida albicans*	1	*Candida albicans*	*C. albicans*
*Streptococcus agalactiae*	1	*Streptococcus agalactiae*	*S. agalactiae*
*Staphylococcus aureus*	1	*Staphylococcus aureus*	*S. aureus*
*Escherichia coli*	1	*Escherichia coli*	*E. coli*
*Enterococcus faecalis*	1	*Enterococcus faecalis*	*E. faecalis*
Vaginal epithelial cells	2	Vaginal epithelial cells VK2/E6E7-ATCC-CRL-2616	VK2/E6E7 VECs
		Primary vaginal epithelial cells	Primary VECs

### Origin and Culture of VECs

Two kinds of VECs, including the VK2/E6E7-ATCC-CRL-2616 (VK2/E6E7) and primary VECs, were used in our study (Table 1). The VK2/E6E7 VECs were obtained from the American Type Culture Collection (ATCC; Rockville, MD, USA). This cell line from the normal vaginal mucosal tissue of a premenopausal woman, who underwent anterior-posterior vaginal repair surgery, was established in 1996 (31). Primary VECs were collected from a healthy volunteer during the 17th and 18th days of her menstrual cycle. The volunteer had not used antibiotics, spermicidal products, or oral contraceptives, and had no known vaginal pathology. The VECs were digested with 0.15% trypsin and 0.01% EDTA for 5 min, and the digestion was terminated using Dulbecco's Modified Eagle's Medium-nutrient mixture F-12 (DMEM-F12) medium that contained 10% fetal bovine serum. Next, the cells were centrifuged for 10 min (13,400 × g), and the supernatant was discarded. The VEC concentration was adjusted to 10^5^ cells/mL using keratinocyte serum-free medium (K-SFM) containing antibiotics (counted by the cell-counting plate method and then diluted to the appropriate concentration). VEC suspensions (2 mL) were pipetted into a 6-well-culture plate with a built-in coverslip and kept for 18 h until adhesion to the cells. The VECs that did not adhere were removed by washing thrice with DMEM-F12 medium (without antibiotics) containing 10% fetal calf serum. Both kinds of adherent VECs in the RPMI1640 medium in each well were used to evaluate the adhesion of the five *Lactobacillus* strains. Only the adherent VK2/E6E7 VECs in each well were used for the adhesion inhibition experiments.

### Adhesion of *Lactobacillus* to the VECs

VK2/E6E7 and primary VECs were used in this experiment. A *Lactobacillus* suspension (2 mL), at a concentration of 1.0 × 10^8^ CFU/mL, was added to the adherent VECs in each well. They were then cultivated at 37°C in an atmosphere of 5% CO_2_ for 1 h. The coverslip was removed, fixed in methanol for 15 min, and subjected to Gram staining. *Lactobacillus* strains were identified based on their color and morphology and counted. *Lactobacillus* adhesion was assessed using the adhesion index, which was calculated as follows: Adhesion index = adhering bacteria/cell number. The adherent *Lactobacillus* number is the total quantity of *Lactobacilli* adherent to 50 intact VECs, which were selected randomly. We observed these intact VECs and counted the *Lactobacillus* number using a light microscope at 1000 × magnification under oil immersion (32).

### *Lactobacillus*-Mediated Inhibition of Pathogen Adhesion to VK2/E6E7 VECs

The results of the *Lactobacillus* adhesion evaluation experiments indicated that the source of the VECs might not affect the selection of the most adhesive *Lactobacillus* strain. The VK2/E6E7 VECs, as a model cell line, ensured the reproducibility of the results of the current experiment. Therefore, it was chosen for evaluating the inhibitory effects of the five *Lactobacillus* strains on pathogen adhesion.

For the adhesion inhibition experiments, the concentration of each bacterium was adjusted to 1.0 × 10^8^ CFU/mL with RPMI1640 broth using a microplate reader. The adhesion inhibition experiments included exclusion, displacement, and competition inhibition experiments. Briefly, the built-in coverslip was removed, and pathogen adhesion was evaluated upon completion of the experiment. Pathogen adhesion to the VK2/E6E7 VECs was assessed using the adhesion index, as described previously (32). The adherent pathogen number is the quantity of pathogens adherent to 50 intact VECs (VK2/E6E7) selected at random. The pathogens that adhered to the VK2/E6E7 VECs because adhesion inhibition by *Lactobacillus* was absent were defined as the control group. The pathogens that adhered to the VK2/E6E7 VECs after *Lactobacillus* antagonization were defined as the experimental group. The inhibitory effects of *Lactobacillus* on pathogen adhesion were negatively correlated with pathogen adhesion to the VK2/E6E7 VECs in the experimental group. In other words, the stronger the pathogen adhesion, the weaker the inhibitory effects of *Lactobacillus* on pathogen adhesion in the experimental group. The statistically significant difference in the pathogen adhesion indexes of the control and experimental groups indicated that *Lactobacillus* showed inhibitory effects on pathogen adhesion.

### Exclusion Experiment

For the control group, the pathogen suspension (1 mL) was added to a well with adherent VK2/E6E7 VECs. For the experimental group, the *Lactobacillus* suspension (1 mL) was added to another well with adherent VK2/E6E7 VECs. They were incubated at 37°C in an atmosphere of 5% CO_2_ for 1 h. The non-adherent *Lactobacillus* and pathogens were removed by washing thrice with sterile phosphate-buffered saline (PBS). The pathogen suspension (1 mL) was added to the wells of the experimental and control groups and then incubated at 37°C in an atmosphere of 5% CO_2_ for an additional hour. Adhesion was then evaluated.

### Displacement Experiment

The pathogen suspension (1 mL) was added to the wells containing adhered VK2/E6E7 VECs of the experimental and control groups. The cells were then incubated at 37°C in an atmosphere of 5% CO_2_ for 1 h. The non-adherent pathogens were removed by washing thrice with sterile PBS. *Lactobacillus* (1 mL) was added to the well of the experimental group, whereas the pathogens (1 mL) were added to that of the control group. The cells were then cultured at 37°C in an atmosphere of 5% CO_2_ for another hour, and adhesion was evaluated.

### Competition Test

For the experimental group, the *Lactobacillus* suspension (1 mL) and the pathogens were mixed and incubated in a well containing adhered VK2/E6E7 VECs. For the control group, the pathogen suspension (1 mL) and RPMI1640 broth (1 mL) were added to the well with the adherent VK2/E6E7 VECs. They were then incubated at 37°C in an atmosphere of 5% CO_2_ for 1 h, and adhesion was evaluated.

### Statistical Analysis

All experimental steps were repeated in triplicate to ensure the reproducibility of the results. The adhesion index values are expressed as the mean ± standard deviation (SD). An independent sample *t*-test was used to statistically analyze the differences between the two groups. A *P* < 0.05 was considered statistically significant. The statistical software used was SPSS 20.0.

### Ethical Approval

The Ethics Committee of Peking University First Hospital approved this study (V2.0/201504.20), and written informed consent was obtained from all participants.

## Results and Discussion

In the current study, five *Lactobacillus* strains, two *L. gasseri* and three *L. crispatus* strains, were isolated from the vagina of healthy women. The adhesion and inhibitory effects of these strains on pathogen adhesion were evaluated.

### Adhesion of *Lactobacillus* to VECs

**Figure 1:**
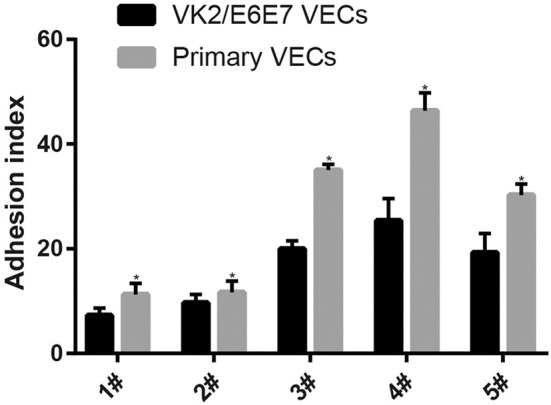
Adhesion of the five *Lactobacillus* strains to VECs. Two kinds of VECs, VK2/E6E7, and primary VECs were used in this experiment. The adhesion of the five *Lactobacillus* strains to the VK2/E6E7 VECs, compared with that to primary VECs, was observed. *Lactobacillus* strains were identified by their color and morphology after Gram staining. *Lactobacillus* adhesion was assessed using the adhesion index: Adhesion index, adhering *Lactobacillus*/cell number. The adhering *Lactobacillus* number is the total quantity of *Lactobacilli* adherent to 50 intact VECs, which were selected at random. These intact VECs were observed, and the *Lactobacillus* number was counted using a light microscope at 1000 × magnification under oil immersion. Each adhesion index value is shown as the mean ± SD. Student's *t*-test was performed. *P < 0.05; the difference in the adhesion of each *Lactobacillus* strain to the VK2/E6E7 and primary VECs was statistically significant.

The adhesion of every *Lactobacillus* strain was stronger in the primary VECs than in the VK2/E6E7 VECs (*P* < 0.05) (Figure 1). These results indicated that the source of the VECs could affect the adhesion of the same *Lactobacillus*. The results could explain the differences in the effectiveness of *Lactobacillus* preparations in different populations (9, 25, 33) and the necessity of clinical trials to verify the anti-pathogenic effects of *Lactobacillus* preparations. The descending order of the five *Lactobacillus* strains based on the strongest adhesion to both types of VECs was 4#, 3#, 5#, 2#, and 1# (Figure 1). The results indicated that the source of the VECs might not affect the selection of the most adhesive *Lactobacillus* strain. VK2/E6E7 VECs, as a model cell line, ensured the reproducibility of the current results. Therefore, it was used in the experiments on the inhibitory effects of *Lactobacillus* on pathogen adhesion.

**Figure 2:**
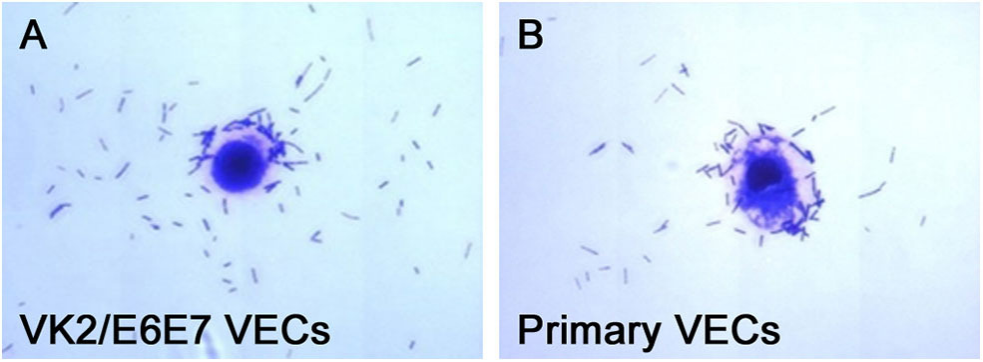
Micrographs of *L. crispatus* 4# adhesion to the VECs. *L. crispatus* 4# adhesion to **(A)** the VK2/E6E7 VECs and **(B)** the primary VECs is shown.

The adhesion of the three *L. crispatus* strains was stronger than that of the two *L. gasseri* strains (*P* < 0.05) (Figure 1), and this result was consistent with that of the study by Mousavi et al. (24). Although Mousavi et al. (24) chose Vero and HeLa cells to investigate *Lactobacillus* adherence, their results suggested that *L. crispatus* adhesion was stronger than *L. gasseri* adhesion, irrespective of the type of cells used. The *L. crispatus* and *L. gasseri* adhesion observed in both our and Mousavi's studies (24) was weaker than the *Lactobacillus fermentum* and *Lactobacillus rhamnosus* adhesion, which was investigated in the study by Ortiz et al. (34). Among the five *Lactobacillus* strains, *L. crispatus* 4# showed the strongest adhesion, and its adhesion indexes for primary and VK2/E6E7 VECs were 46.4 ± 3.41 and 25.4 ± 4.2, respectively (Figure 2). The differences in the adhesion of the *Lactobacillus* strains could be attributed to the different sizes, species, and growth rates of *Lactobacilli* and the different adherent cell types (24, 34, 35). Additionally, *Lactobacillus* adhesion was positively related to the secretion of a protein that contained MucBP-like domains (N506_1778) and a putative novel adhesin (N506_1709) with rib/alpha-like domain repeats and negatively related to the production of exopolysaccharides (36).

Adhesion not only helps *Lactobacillus* protect the mucosal epithelium but is also an important virulence factor of pathogens, beneficial for colonization, and biofilm formation (37). It is difficult for single-use antibiotics to penetrate biofilms (38); thus, biofilms protect pathogens and promote disease recurrence (39–41).

### Effects of *Lactobacillus* on Pathogen Adhesion

**Figure 3:**
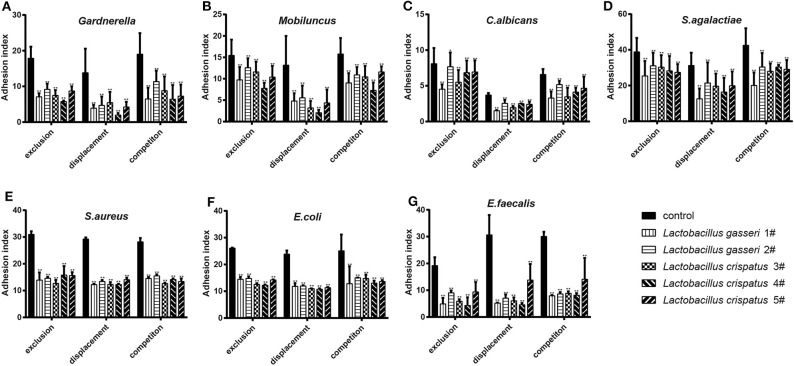
Inhibitory effects of the five *Lactobacillus* strains on the adhesion of seven pathogenic strains to the VK2/E6E7 VECs. **(A–G)** The adhesion indexes of each pathogen in the control and experimental groups are shown. The control group shows the adhesion indexes of the pathogens when their adhesion was not inhibited by *Lactobacillus* (1#, 2#, 3#, 4#, and 5#), whereas the experimental groups show the adhesion indexes of each pathogen after the exclusion, displacement, and competition effects of *Lactobacillus* antagonization. Adhesion index, adhering pathogen/cell number. The adhering pathogen number is the total quantity of pathogens adherent to 50 intact VECs, which were selected at random. These intact VECs were observed, and the pathogen number was counted using a light microscope at 1000 × magnification under oil immersion. Each adhesion index value is shown as the mean ± SD. Student's *t*-test was performed. *P < 0.05; **P <0.001; the difference in the adhesion indexes of the pathogens in the control and experimental groups was statistically significant.

**Figure 4:**
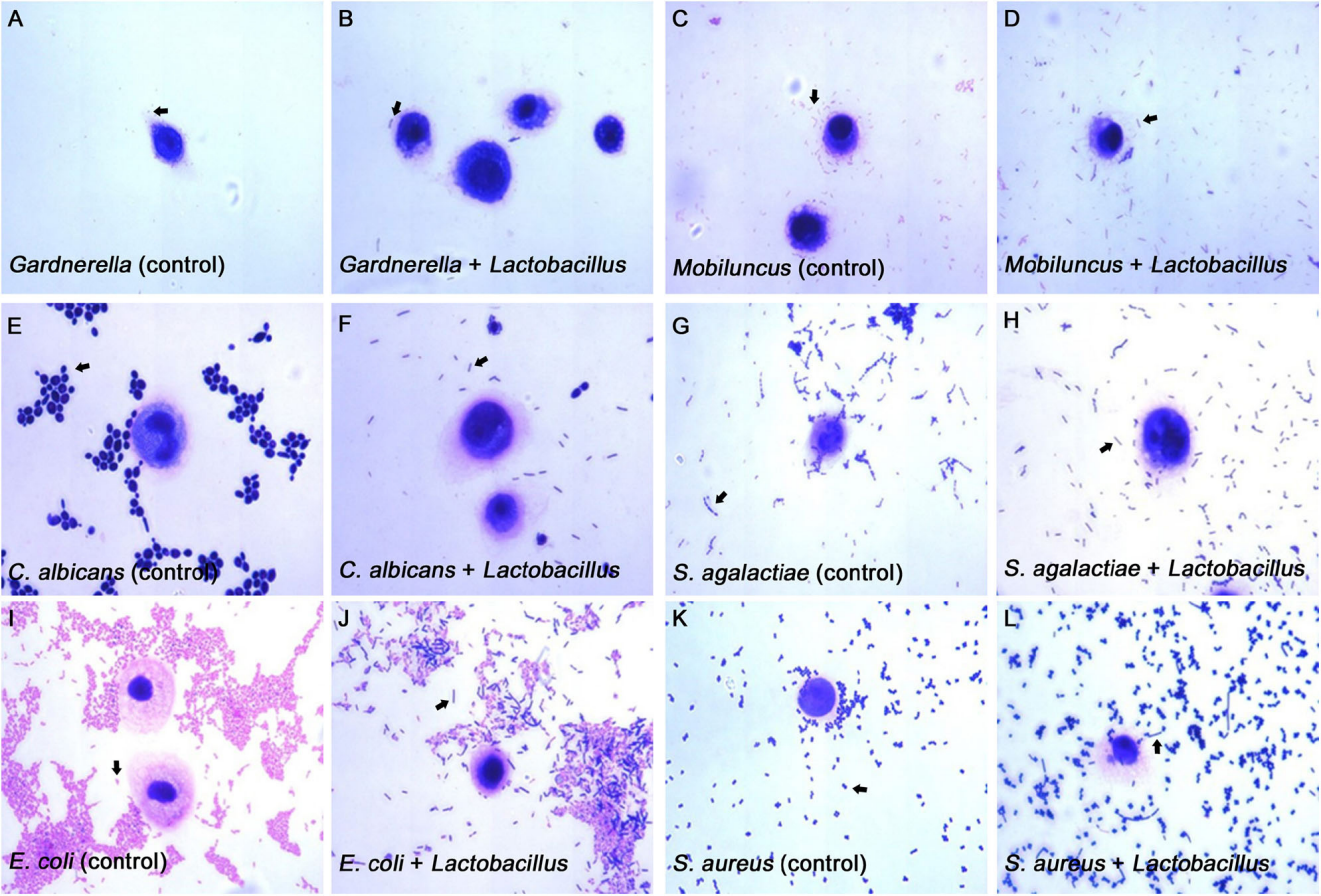
Micrographs of the inhibitory effects of *Lactobacillus* on pathogen adhesion to the VK2/E6E7 VECs. **(A–L)** These micrographs illustrate the pathogen adhesion to the VK2/E6E7 VECs in the control and experimental groups. **(A,C,E,G,I,K)**. These micrographs illustrate pathogen adhesion to the VK2/E6E7 VECs in each control group. Both *Lactobacillus* and pathogens were identified by their color and morphology after Gram staining. In panel **(A)**, the arrow refers to *Gardnerella*, which appears as a red short rod. In panel **(C)**, the arrow refers to *Mobiluncus*, which appears as a red curved rod. In panel **(E)**, the arrow refers to *C. albicans*, which appears as a purple sphere or oval. In panel **(N)**, the arrow refers to *S. agalactiae*, which appears as a purple chain spheroid. In panel **(I)**, the arrow refers to *E. coli*, which appears as a red rod. In panel **(K)**, the arrow refers to *S. aureus*, which appears as a purple globule. **(B,D,F,H,J,L)** These micrographs illustrate both the pathogens and *Lactobacillus* that adhere to the VK2/E6E7 VECs after *Lactobacillus* antagonization. In panel **(B,D,F,H,J,L)**, the arrows refer to *Lactobacillus*, which appears as a purple long rod.

When the *Lactobacillus*-mediated antagonization of *Gardnerella* adhesion to the VK2/E6E7 VECs was tested, the descending orders of the strains with the strongest inhibition were 4#, 1#, 3#, 5#, and 2# in the exclusion experiments; 4#, 1#, 5#, 2#, and 3# in the displacement experiments; and 4#, 1#, 5#, 3#, and 2# in the competition experiments (Figure 3A). These results suggested that, among the five *Lactobacillus* strains, *L. crispatus* 4# showed the strongest inhibition of *Gardnerella* adhesion. Similarly, *L. crispatus* 4# also showed the strongest inhibition of *Mobiluncus* (Figure 3B), *E. coli* (Figure 3F), and *E. faecalis* (Figure 3G) adhesion. *L. crispatus* 3# showed the strongest inhibition of *S. aureus* adhesion (Figure 3E). The descending orders of the strains with the strongest inhibitory effects on *S. aureus* were 3#, 1#, 2#, 5#, and 4# in the exclusion experiments; 1#, 3#, 4#, 2#, and 5# in the displacement experiments; and 3#, 5#, 4#, 1#, and 2# in the competition experiments (Figure 3E). *L. gasseri* 1# showed the strongest inhibition of *C. albicans* (Figure 3C) and *S. agalactiae* (Figure 3D) adhesion. The descending orders of the strains with the strongest inhibitory effects on *S. agalactiae* adhesion were 1#, 5#, 4#, 3#, and 2# in the exclusion experiments; 1#, 4#, 3#, 5#, and 2# in the displacement experiments; and 1#, 3#, 5#, 4#, and 2# in the competition experiments (Figure 3D). However, further investigation of the antibacterial properties of *Lactobacillus* and clinical trials are required to confirm these results. More details are shown in Figures 3, 4.

All five *Lactobacillus* strains could effectively antagonize pathogen adhesion to the VK2/E6E7 VECs through exclusion, displacement, and competition inhibition (*P* < 0.05, Figure 3). However, the degree of the inhibitory effects of these strains on pathogen adhesion was not consistent. The current observations partially elucidated the mechanisms by which *Lactobacillus* preparations successfully antagonized pathogenic infections in clinical trials and showed that these effects were different (9, 25, 33).

*Lactobacillus* effectively prevented pathogen adhesion to the VECs (*P* < 0.05, Figure 3), indicating that *Lactobacillus* adhesion to VECs was beneficial for the prevention of pathogenic infections. For each *Lactobacillus* strain, the displacement effect was stronger than the exclusion and competition effects on pathogen adhesion (Figure 3). This showed that *Lactobacillus* could effectively remove pathogens attached to the vaginal epithelium; thus, the results provide a basis for the preparation of *Lactobacillus* formulations for effective vaginal infection treatment. Multiple studies have shown that exopolysaccharides produced by *Lactobacillus* can shield adhesin and reduce pathogen adhesion to cell surfaces (42–44). Ortiz et al. (34) selected *L. fermentum* and *L. rhamnosus* to antagonize *S. aureus* and *S. agalactiae* adhesion to VECs; they found that the competition effect was far stronger than the exclusion and displacement effects. Abedi et al. (45) showed that the exclusion, displacement, and competition effects were not significantly different. These findings suggested that *Lactobacillus* could effectively prevent pathogen adhesion to VECs, but the effects of this adhesion inhibition varied; possible reasons for this include differences in the anti-adhesive substances produced when different *Lactobacillus* species compete for the same adhesion receptor (46). Anti-adhesive substances can degrade pathogenic carbohydrate receptors, construct biofilms, induce biosurfactant production, produce receptor analogs, and cause steric blockade or receptor inhibition (45, 47, 48). Other possible causes include the time and initial amount of the pathogens in the *in vitro* culture (49), the bacterial size, and the presence of the *arcA* gene in the bacteria (48).

## Conclusion

We found that the origin of the VECs might not affect the selection of the most adhesive *Lactobacillus* strains. *L. crispatus* showed stronger adhesion to the VECs than *L. gasseri*. The results of the exclusion, displacement, and competition inhibition experiments indicated that *L. gasseri* 1# could be used as a suitable probiotic for the prevention of *C. albicans* and *S. agalactiae* infections, whereas *L. crispatus* 3# could be used as a suitable probiotic against *S. aureus* infections. *L. crispatus* 4# was found to be promising for the prevention of *Gardnerella, Mobiluncus, E. coli*, and *E. faecalis* infections. However, further research on the antibacterial properties of *Lactobacillus*, followed by clinical validation, is required.

## Data Availability Statement

All datasets generated for this study are included in the article/supplementary material.

## Ethics Statement

The studies involving human participants were reviewed and approved by Ethics committee of Peking University first hospital. The patients/participants provided their written informed consent to participate in this study.

## Author Contributions

YH, XN, BW, RN, BX, and HY conceived the study design. XN, BW, and RN were responsible for the recruitment of volunteers and the collection of samples. YH, XN, and RN performed the laboratory assays. YH and BW performed the data analysis, and YH wrote the initial manuscript. BX and HY revised the manuscript. All the authors read and approved the final version of the manuscript.

## Conflict of Interest

The authors declare that the research was conducted in the absence of any commercial or financial relationships that could be construed as a potential conflict of interest.
